# Transcriptome Profiling of Taproot Reveals Complex Regulatory Networks during Taproot Thickening in Radish (*Raphanus sativus* L.)

**DOI:** 10.3389/fpls.2016.01210

**Published:** 2016-08-22

**Authors:** Rugang Yu, Jing Wang, Liang Xu, Yan Wang, Ronghua Wang, Xianwen Zhu, Xiaochuan Sun, Xiaobo Luo, Yang Xie, Muleke Everlyne, Liwang Liu

**Affiliations:** ^1^National Key Laboratory of Crop Genetics and Germplasm Enhancement, College of Horticulture, Nanjing Agricultural UniversityNanjing, China; ^2^School of Life Science, Huaibei Normal UniversityHuaibei, China; ^3^Department of Plant Sciences, North Dakota State UniversityFargo, ND, USA

**Keywords:** *Raphanus sativus* L., taproot, thickening, digital gene expression, RNA-Seq

## Abstract

Radish (*Raphanus sativus* L.) is one of the most important vegetable crops worldwide. Taproot thickening represents a critical developmental period that determines yield and quality in radish life cycle. To isolate differentially expressed genes (DGEs) involved in radish taproot thickening process and explore the molecular mechanism underlying taproot development, three cDNA libraries from radish taproot collected at pre-cortex splitting stage (L1), cortex splitting stage (L2), and expanding stage (L3) were constructed and sequenced by RNA-Seq technology. More than seven million clean reads were obtained from the three libraries, from which 4,717,617 (L1, 65.35%), 4,809,588 (L2, 68.24%) and 4,973,745 (L3, 69.45%) reads were matched to the radish reference genes, respectively. A total of 85,939 transcripts were generated from three libraries, from which 10,450, 12,325, and 7392 differentially expressed transcripts (DETs) were detected in L1 vs. L2, L1 vs. L3, and L2 vs. L3 comparisons, respectively. Gene Ontology and pathway analysis showed that many DEGs, including *EXPA9, Cyclin, CaM, Syntaxin, MADS-box, SAUR*, and *CalS* were involved in cell events, cell wall modification, regulation of plant hormone levels, signal transduction and metabolisms, which may relate to taproot thickening. Furthermore, the integrated analysis of mRNA-miRNA revealed that 43 miRNAs and 92 genes formed 114 miRNA-target mRNA pairs were co-expressed, and three miRNA-target regulatory networks of taproot were constructed from different libraries. Finally, the expression patterns of 16 selected genes were confirmed using RT-qPCR analysis. A hypothetical model of genetic regulatory network associated with taproot thickening in radish was put forward. The taproot formation of radish is mainly attributed to cell differentiation, division and expansion, which are regulated and promoted by certain specific signal transduction pathways and metabolism processes. These results could provide new insights into the complex molecular mechanism underlying taproot thickening and facilitate genetic improvement of taproot in radish.

## Introduction

Radish (*Raphanus sativus* L., 2n = 2x = 18) belonging to the Brassicaceae family, is an important root vegetable crop planted all over the world. The fleshy taproot comprises main edible portion of the plant with high nutrition and medical value, and is rich in carbohydrate, folic acid, ascorbic acid and sulforaphane (Chaturvedi, 2008). Taproot thickening of radish is a complex biological process involving morphogenesis and dry matter accumulation. Thus, understanding the regulatory mechanism of taproot thickening is important for improving the yield and quality of radish.

In the past decades, the thickening mechanism of taproot has been extensively studied anatomically and physiologically. The thickening taproot is comprised of the hypocotyl and root axis, and is mainly driven by parenchyma cell division and subsequent cell expansion in the cambium, which produces a substantial core of secondary xylem and a slightly broader secondary phloem (Tsuro et al., 2008). The development of cortex splitting is an important sign of the initiation of thickening growth of taproot in radish. Additionally, some physiological studies revealed that many hormones or environmental factors could affect taproot thickening (Matveeva et al., 2004; Choi et al., 2011). For example, the involvement of cytokinin, gibberellic acid (GA), indole acietic acid (IAA), abscisic acid (ABA) and ethylene, in taproot formation has been investigated in radish (Matveeva et al., 2004; Jung and McCouch, 2013). However, root formation and response to the environment are essential results of selective expression of related genes (Petricka et al., 2012). Recently, some genes involved in regulating storage root formation have been identified in some plant species (You et al., 2003; Tanaka et al., 2005; Ku et al., 2008). In sweet potato, Tanaka et al. (2005) reported that a receptor-like kinase gene (*RLK*) (similar to Leucine-rich repeat (LRR) II RLK family) was highly expressed in the primary cambium and meristems of the xylem, which is the site for actively diving cells and causes thickening of storage roots. MADS-box 1 gene was also found to be involved in the initiation and development of storage roots by triggering plant hormones jasmonic acid and cytokinin (Ku et al., 2008). Nevertheless, the isolation of key genes associated with radish taproot thickening remains to be limited to date.

With the development next-generation sequencing (NGS) technology, RNA-seq has now been the preferred method for gene expression profiling (McGettigan, 2013). RNA-seq can provide digital gene expression (DGE) measurement (Wang et al., 2010). Recently, DGE tag profiling has been used to identify differentially expressed genes (DEGs) in different tissues, organs and developmental stages in plants (Li et al., 2011; Cheng et al., 2013; Park et al., 2014; Zhang et al., 2014a,c). Using this approach, several studies have been identified many DEGs and explored the roles of DEGs in root development in maize (Li et al., 2011), cucumber (Zhang et al., 2014a), Brassica (Zhang et al., 2014c) and Lotus (Cheng et al., 2013). Recently, the genome sequencing and root transcriptomics studies have provided useful platform for comprehensive investigation of the molecular mechanisms in radish taproot development (Wang et al., 2012, 2013a; Kitashiba et al., 2014). However, no studies on identification of DEGs and their roles in regulating taproot growth and thickening have been conducted in radish. In this study, to identify and analyze the global gene expression data during radish taproot thickening, three cDNA libraries prepared from pre-cortex splitting stage (L1: 10 DAS), cortex splitting stage (L2: 20 DAS) and expanding stage (L3: 40DAS) were sequenced by Solexa/Illumina HiSeq™ 2500 platform. Furthermore, based on association analysis between taproot thickening-related DEGs and miRNAs, the schematic model of regulatory networks associated with radish taproot thickening was proposed. These results could facilitate revealing the complex gene regulatory networks of radish taproot thickening, and provide novel insights into the molecular mechanism underlying storage root development in radish.

## Materials and methods

### Plant material

The radish (*Raphanus sativus* L.) advanced inbred line ‘NAU-YH’ was used in this study. Surface-sterilized seeds were germinated on a moist filter paper in darkness for 3 days, and then grown in plastic pots with a mixture of soil and peat substrate (1:1, V/V) in greenhouse at 25°C/18°C (day/night). In addition, the development of cortex splitting is an important signal of the initiation of taproot thickening growth in radish due to the cortex cells cannot divide and expand (Wang et al., 2012). The root cortex split was initiated at about 12 days after sowing (DAS), and the full root cortex splitting was achieved over a period of 22 DAS. The taproot thickening growth was rapidly expanded from the 22 to 42 DAS. Based on the time point of different developmental stages, samples of taproots were harvested at three different developmental stages: pre-cortex splitting stage (L1, 10 DAS), cortex splitting stage (L2, 20 DAS) and expanding stage (L3, 40 DAS). The subsamples of taproots were collected from four developmental stages (10, 20, 40 and 50 DAS) for RT-qPCR analysis. At least three independent biological replicates for taproot sample/subsample of each stage were collected. All harvested tissues were immediately frozen in liquid nitrogen and stored at −80°C until use.

### RNA sequencing library construction and illumina sequencing

Total RNA of the independent taproots from three different stages were independently isolated using Trizol® Reagent (Invitrogen, USA) according to the manufacturer's protocol, and treated with RNase-free DNase I (Takara, Japan) to degrade DNA contamination. Then, the three independently isolated total RNA samples of each stage were equally pooled into one RNA sample, which was used for library preparation and sequencing. Three RNA sequencing libraries (L1, L2 and L3) were constructed and sequenced using Illumina HiSeq™ 2500 platform at the Beijing Genomics Institute (BGI, Shenzhen, China). The library construction and Illumina sequencing were performed according to a previously described method (Cheng et al., 2013).

### Identification and functional annotation of differentially expressed genes

By base calling, the raw reads was filtered to remove adaptor sequences, low quality reads and empty reads. Subsequently, the clean reads were mapped to the radish reference sequences containing genomic survey sequences (GSSs, http://www.ncbi.nlm.nih.gov/nucgss/?term=radish), expressed sequence tags (ESTs, http://www.ncbi.nlm.nih.gov/nucest/?term=radish), and a ‘NAU-YH’ radish root mRNA transcriptome [Sequence Read Archive (SRA) accession No.SRX707630] using SOAP2 software (Li et al., 2009). No more than two mismatches were allowed in the alignment.

To obtain statistical confirmation of gene expression among three radish taproot libraries, the gene expression level in each library was normalized using RPKM method (Reads Per kb per Million reads) (Mortazavi et al., 2008), and those uniquely matched reads were used for calculating the genes RPKM values. Subsequently, the differential expression detection of genes across libraries was conducted using a strict algorithm method (Audic and Claverie, 1997). The absolute value of |log_2_ Ratio|≥ 1 with false discovery rates (FDR) ≥ 0.001 and *P* < 0.005 was set as the threshold to judge the significance of differences in gene expression across libraries. Furthermore, the DEGs were mapped to Gene Ontology (GO) database (http://www.geneontology.org/) and Kyoto Encyclopedia of Genes and Genomes (KEGG) database to identify significantly enriched functional classification and metabolic pathways. The Blast2GO program (http://www.blast2go.com/) and WEGO software were used for GO annotation and functional classification of DEGs, respectively (Ye et al., 2006).

### Association analysis of mRNA and microRNA

To investigate the mRNA and microRNA involved in the thickening of radish taproot, an integrated analysis of mRNA-miRNA using NGS was performed. Briefly, mRNAs and microRNAs libraries were sequenced from the same samples using Illumina HiSeq system. Then, the normalized miRNA and mRNA data was used to analyze miRNA and mRNA expression, and identify the differentially expressed miRNAs (DEmiRs) and mRNAs (DEGs) across libraries (|log_2_Ratio| ≥ 1). The DEmiRs and DEGs were identified with a threshold of |log_2_Ratio| ≥ 1. Subsequently, the targets of each miRNA were predicted based on sequence complementarity with mRNA sequences. Next, the correlation analysis between mRNA and miRNA expression were performed, and a contingency table was created for all DEmiRs and DEGs by removing the unreliable miRNA-mRNA pairs. Finally, a miRNA-target gene regulatory network was constructed across libraries using Cytoscape_v3. 2. 1 program for illustrating identified differentially expressed miRNA-mRNA target networks. The miRNAs sequencing data during radish taproot thickening were obtained from our previous study (Yu et al., 2015).

### Expression analysis of differentially expressed genes using RT-qPCR

Total RNAs were extracted from the taproot samples (10, 20, 40, and 50 DAS) using Trizol® Reagent (Invitrogen, USA) and then reverse transcribed into cDNA with PrimeScript® RT reagent Kit (Takara, Dalian, China). RT-qPCR was carried out using SYBR Premix Ex Taq™ II (Takara, Dalian, China) on an iCycler IQ real-time PCR detection system (BIO-RAD) according to previous methods (Wang et al., 2013b). Each sample had three biological replicates with three technical replicates for each of the biological replicate. The relative expression level was calculated by the equation ratio 2^−Δ*ΔCτ*^. The primers of selected genes were designed using Beacon Designer 7.0 software (Table S1), and *Actin* gene was used as the internal control.

## Results

### Digital gene expression sequencing and data analysis

**Figure 1:**
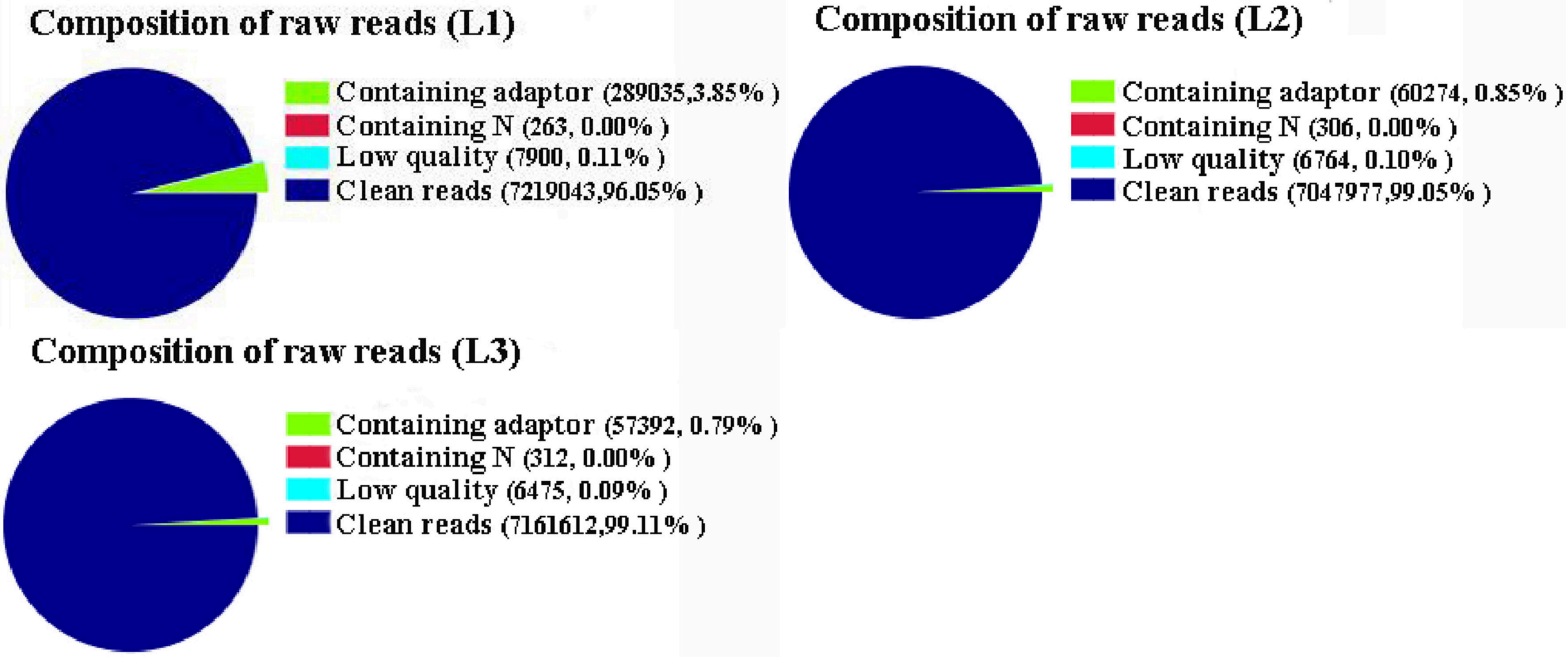
**The composition of raw reads in three libraries**. L1: pre-cortex splitting; L2: cortex splitting stage; L3: expanding stage.

To investigate genes involved in radish taproot thickening, three RNA-seq libraries including L1 (pre-cortex splitting stage), L2 (cortex splitting stage) and L3 (expanding stage) libraries were performed by Illumina HiSeq™ 2500 platform. Based on Illumina sequencing, a total of 7,516,241 (L1), 7,115,321 (L2), and 7,225,791 (L3) raw reads were generated. After discarding the adaptor sequences and low-quality reads, 7,219,043, 7,047,977, and 7,161,612 clean reads were obtained in three libraries, respectively (Figure 1). The clean reads were then mapped to the preparing reference genome. A large proportion of clean reads (86.71, 88.51 and 88.70% in L1, L2 and L3 libraries, respectively) were mapped to the reference genes (Table 1). Among these clean reads, 48.47% mapped to unique genes, and the uniquely matched reads were used for gene expression analysis of each library.

**Table 1:** **Summary of alignment statistics of RNA-Seq in three libraries mapped to reference genome**.

**Summary**	**L1**		**L2**		**L3**	
	**Reads number**	**Percentage**	**Reads number**	**Percentage**	**Reads number**	**Percentage**
Total clean reads	7219043	100.00%	7047977	100.00%	7161612	100.00%
Total base pairs	353733107	100.00%	345350873	100.00%	350918988	100.00%
Total mapped reads	6259457	86.71%	6238129	88.51%	6352466	88.70%
Perfect match	4717617	65.35%	4809588	68.24%	4973745	69.45%
≤ 2bp mismatch	1541840	21.36%	1428541	20.27%	1378721	19.25%
Unique match	3235230	44.82%	3427832	48.64%	3719999	51.94%
Multi-position match	3024227	41.89%	2810297	39.87%	2632467	36.76%
Unmapped reads	959586	13.29%	809848	11.49%	809146	11.30%

### The expression of all detected transcripts in each of the three stages

**Figure 2:**
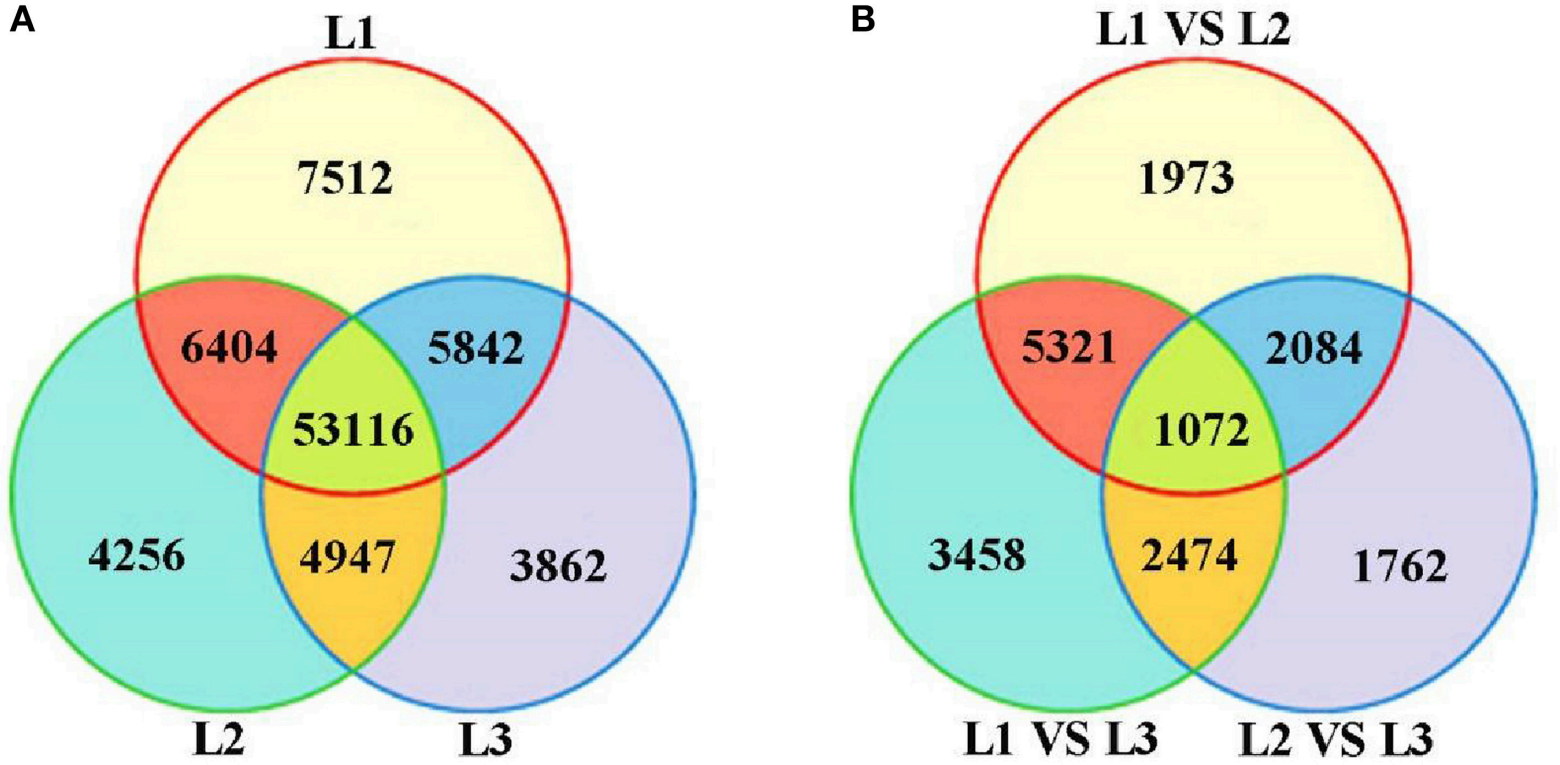
**The all detected gene expression (A) and differential gene expression (B) showed in Venn diagram**. L1 vs. L2: pre-cortex splitting vs. cortex splitting stage; L1 vs. L3: pre-cortex splitting vs. expanding stage; L2 vs. L3: cortex splitting vs. expanding stage.

Based on deep sequencing of the three cDNA libraries, a total of 85,939 transcripts were generated during radish taproot thickening. Of these, 72,874 (L1), 68,723 (L2) and 67,767 (L3) transcripts were detected in three libraries. A Venn diagram shows the number of uniquely expressed transcripts in each library and transcripts that were shared between one or more other libraries (Figure 2A). Of these, 53,116 transcripts were shared by all three DGE libraries, and 15,630 transcripts (7512, 4256 and 3862 in the L1, L2 and L3 libraries, respectively) were specifically expressed in a single library. Additionally, the number of transcripts exclusively expressed in two taproot thickening stages was also compared. The number of transcripts synchronously expressed in pre-cortex splitting stage and cortex splitting stage was more than that expressed synchronously in the other two taproot thickening stages. These dynamic expressions of co-expressed transcripts suggest that they might determine the changing of radish taproot thickening, and the specifically expressed transcripts suggested that they might play a vital role at the corresponding stage. The statistical analysis of identified transcripts among the three libraries is shown in Figure 2A.

### Identification of differentially expressed transcripts

**Figure 3:**
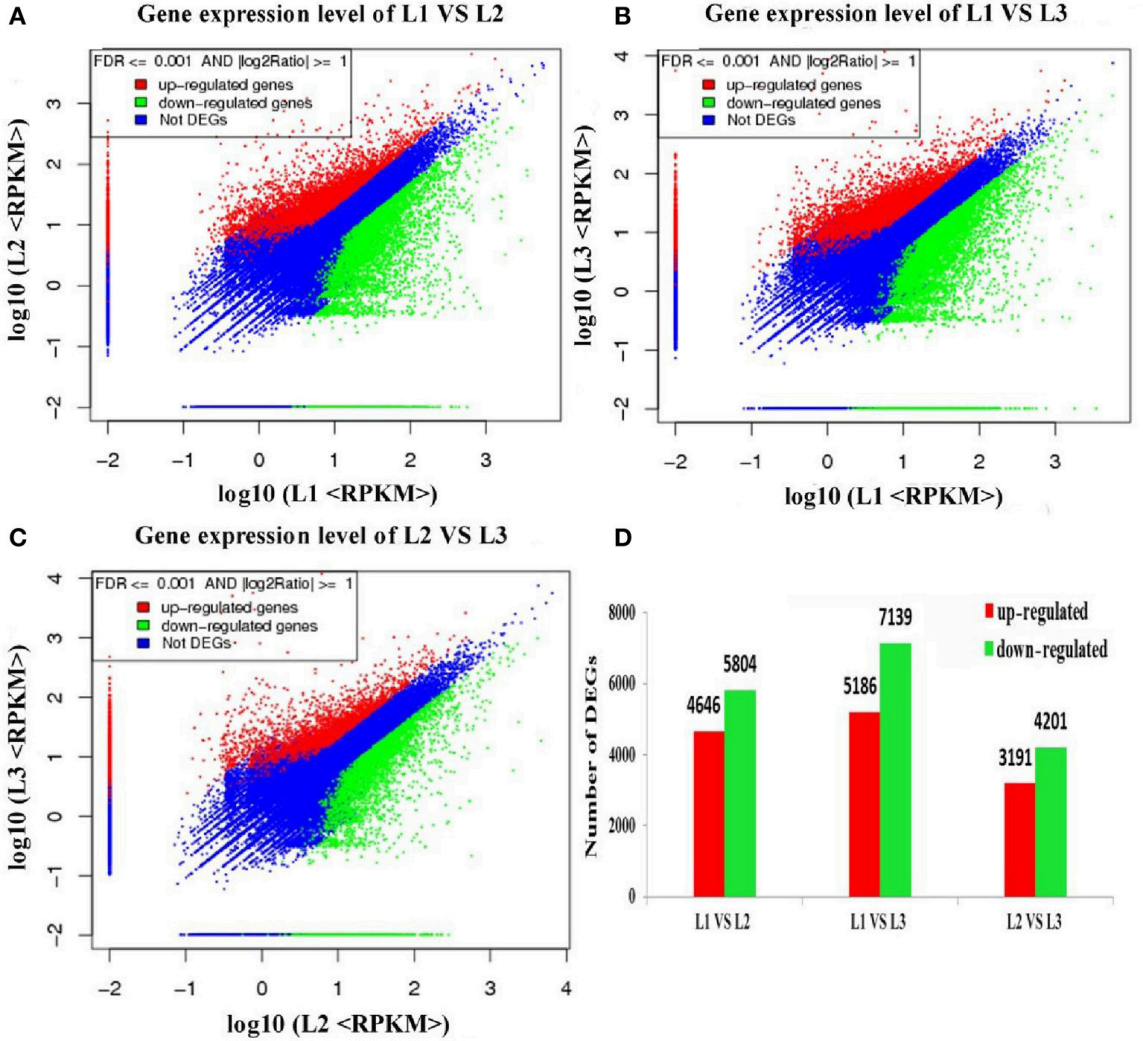
**Differentially expressed genes across all libraries. (A–C)** Scatter plot showing the genes expression levels at pre-cortex splitting, cortex splitting and expanding stage, respectively. **(D)** The numbers of differentially expressed genes in each comparison (L1 vs. L2, L1 vs. L3, L2 vs. L3).

Differential expression analysis was performed to identify differentially expressed transcripts (DETs) during radish taproot thickening. The significant DETs were identified based on a threshold of absolute value of |log_2_ Ratio| ≥ 1 with FDR < 0.001. A total of 18,144 transcripts were found to portray significant differential expression including 16,045 annotated transcripts across libraries (Table S2). The analysis of up- and down-regulated transcripts by scatter plot is shown in Figure 3. A total of 10,450, 12,325 and 7392 transcripts with significant differential expression were found in L1 vs. L2, L1 vs. L3 and L2 vs. L3 comparisons respectively. Of these, 4646, 5186, and 3191 DETs were significantly up-regulated, and 5804, 7139, and 401 DETs were down-regulated in L1 vs. L2, L1 vs. L3, and L2 vs. L3 comparisons respectively (Figure 3D). Meanwhile, as shown in Figure 2B, the largest difference of the number of DETs was found in L1 vs. L3 comparison, while the smallest difference of the number of DETs was found in L2 vs. L3 comparison. Moreover, 17,241 out of 18,144 DETs were successive expressed in at least two libraries. In contrast, only 615, 149, and 140 DETs were identified as stage-specific expression in L1, L2 and L3 library, respectively (Table S2). The change of gene expression was down or up-regulated, perhaps reflecting the molecular mechanisms that control the radish taproot thickening process.

### High expression level of DETs in three comparisons

In order to obtain a general statistical overview of transcriptional gene regulation during radish taproot thickening, the high expression level of DETs (an RPKM value ≥ 100 in at least one of the three comparisons) that were up-and down-regulated in three comparisons is listed in Table S3. From the results, a total of 1049 transcripts that represent about 6% of the total significantly DETs were assessed. The high expression levels of up-regulated DETs in all three comparisons were considered (145 transcripts). Most of these DETs were involved in cellular processes (cellulose synthase, cell division cycle 5-like protein and beta-amylase 3), metabolic processes (sucrose phosphate synthase, sucrose synthase, xyloglucosyl transferase, and ATP sulfurylase), synthesis and transport of matter (sucrose synthase, H(+)-ATPase 1, glucose 6-Pi/Pi transporter), and swelling related proteins (extensin-like protein, enhancer of mRNA-decapping protein 4). Contrasting the up-regulated DETs, the high expression level of down regulated DETs were also observed in three comparisons (305 transcripts); some of them were involved in hormone synthesis and response (SAUR-like auxin-responsive protein, ethylene responsive element binding factor 1, jacalin lectin family protein and ACC oxidase), metabolic processes (chalcone synthase 1 protein, glutamine synthetase and oxidoreductase) and cell proliferation (ARGOS-like protein). All these results suggested that the differentially expressed transcripts might play crucial regulatory roles in the taproot thickening process.

### GO terms and KEGG pathway annotation of differentially expressed transcripts

**Figure 4:**
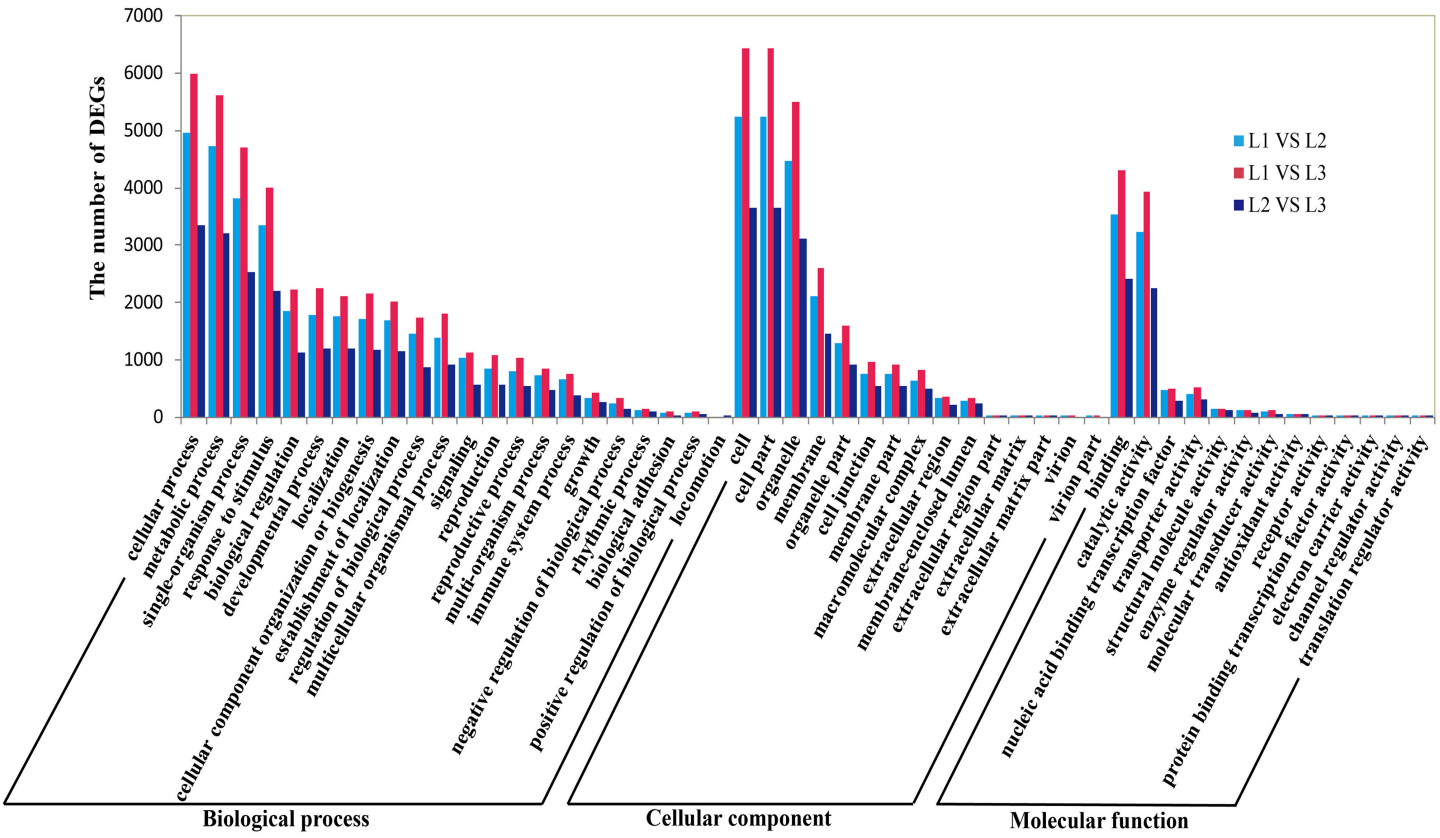
**Gene Ontology (GO) functional enrichment of differentially expressed genes during radish taproot thickening**.

To further establish the main biological function of DETs during radish taproot thickening, the functional annotation was performed by mapping all DETs to Gene Ontology (GO) terms in GO database (http://www.geneontology.org/). A total of 18,144 DETs were categorized into 50 functional groups consisted of 22 biological process, 15 cellular component and 13 molecular function subcategories in pair comparison of each libraries (Figure 4). Among the 22 biological processes, the predominant categories were “cellular process”, “metabolic process”, “single-organism process”, “response to stimulus”, “biological regulation” and “developmental process”, implying that some important metabolic activities and cellular events could involve in radish taproot thickening during all three stages. In addition, GO enrichment analysis was implemented using a Bonferroni-corrected *p* ≤ 0.05 as the threshold. Based on this criterion, 163, 236 and 70 significantly enriched GO terms grouped into three main categories (biological process, cellular component and molecular function) were obtained in three comparisons, respectively (Table S4A). Meanwhile, the most enriched terms (corrected *p* ≤ 0.001) are also screened in pair comparison of each libraries (Table S4B). Among the most enriched GO terms, L1 vs. L2, “single-organism process” (63.6%; GO:0044699) and “response to stimulus” (56.2%; GO:0050896) were the most abundant; L1 vs. L3, “binding” (68.8%, GO:0005488) and “single-organism process” (65.2%; GO:0044699) were the most abundant; L2 vs. L3, “response to stimulus” (56.2%; GO:0050896) and “response to chemical stimulus” (33.3%, GO:0042221) were the most abundant. Moreover, the results also showed a number of significantly enriched genes involved in plant growth and development processes, such as tissue development (GO:0009888), cell growth (GO:0016049), root epidermal cell differentiation (GO:0010053), root development (GO:0048364), root morphogenesis (GO:0010015) and root system development (GO:0022622) (Table S4A).

To further identify metabolic or signal transduction pathways in which the DETs are likely to be involved during radish taproot thickening, pathway enrichment analysis were performed using KEGG database (http://www.genome.ad.jp/kegg/). A total of 5286 (L1 vs. L2), 6217 (L1 vs. L3), and 3666 (L2 vs. L3) DETs were respectively assigned to 127, 126, and 122 pathways by KEGG pathway enrichment analysis in pair comparison of each libraries (Table S5). Among these pathways, metabolic pathway was the largest category (1369, 25.9%; 1601, 25.75%; 874, 23.84%), followed by biosynthesis of secondary metabolites (754, 14.26%; 895, 14.4%; 458, 12.49%), and plant hormone signal transduction (402, 7.6%; 421, 6.77%; 217, 5.92%). Furthermore, 15 (L1 vs. L2 and L2 vs. L3, respectively) and 21 (L1 vs. L3) pathways were identified as significantly enriched (*Q* ≤ 0.05) (Table 2). Of these significantly enriched pathways, the top three of them were similar in the three comparisons including plant hormone signal transduction (ko04075), plant-pathogen interaction (ko04626) and starch and sucrose metabolism (ko00500). The results suggest that genes involved in regulation of plant hormone levels, several metabolism and signal transduction played vital roles in taproot thickening of radish.

**Table 2:** **List of enriched pathways for DEGs in three libraries based on pairwise comparison**.

**Pathway**	**DEGs with pathway annotation**			***Q*-value**			**Pathway ID**
	**L1 vs. L2**	**L1 vs. L3**	**L2 vs. L3**	**L1 vs. L2**	**L1 vs. L3**	**L2 vs. L3**	
Plant hormone signal transduction	402 (7.6%)	421 (6.77%)	217 (5.92%)	0.0000	0.00000	0.00263	ko04075
Plant-pathogen interaction	399 (7.55%)	395 (6.35%)	217 (5.92%)	0.0000	0.00000	0.00128	ko04626
Starch and sucrose metabolism	202 (3.82%)	241 (3.88%)	176 (4.8%)	0.0000	0.00000	0.00000	ko00500
Purine metabolism	114 (2.16%)			0.0062			ko00230
ABC transporters	78 (1.48%)	84 (1.35%)	50 (1.36%)	0.0000	0.00003	0.00263	ko02010
Stilbenoid, diarylheptanoid, and gingerol biosynthesis	77 (1.46%)	94 (1.51%)		0.0020	0.00016		ko00945
Alpha-Linolenic acid metabolism	55 (1.04%)	53 (0.85%)		0.0000	0.00490		ko00592
Sulfur metabolism	51 (0.96%)			0.0001			ko00920
Tryptophan metabolism	50 (0.95%)	66 (1.06%)		0.0399	0.00063		ko00380
Phenylalanine, tyrosine, and tryptophan biosynthesis	47 (0.89%)	51 (0.82%)		0.0014	0.00396		ko00400
Glucosinolate biosynthesis	44 (0.83%)	44 (0.71%)		0.0001	0.00212		ko00966
Ether lipid metabolism	32 (0.61%)			0.0003			ko00565
Isoquinoline alkaloid biosynthesis	27 (0.51%)			0.0020			ko00950
Selenocompound metabolism	23 (0.44%)	30 (0.48%)		0.0004	0.00001		ko00450
Linoleic acid metabolism	18 (0.34%)	15 (0.24%)		0.0003	0.02561		ko00591
Protein processing in endoplasmic reticulum		207 (3.33%)	118 (3.22%)		0.00029	0.03460	ko04141
Phenylpropanoid biosynthesis		174 (2.8%)			0.02735		ko00940
Spliceosome		162 (2.61%)	117 (3.19%)		0.02561	0.00034	ko03040
Cysteine and methionine metabolism		100 (1.61%)			0.01387		ko00270
Endocytosis		94 (1.51%)			0.02195		ko04144
Limonene and pinene degradation		67 (1.08%)			0.00363		ko00903
Inositol phosphate metabolism		60 (0.97%)			0.03047		ko00562
Sulfur metabolism		55 (0.88%)			0.00029		ko00920
Fatty acid metabolism		46 (0.74%)			0.04031		ko00071
Lysine degradation		33 (0.53%)	18 (0.49%)		0.00007	0.01880	ko00310
Ribosome biogenesis in eukaryotes			97 (2.65%)			0.00004	ko03008
Pentose and glucuronate interconversions			83 (2.26%)			0.01425	ko00040
Circadian rhythm - plant			61 (1.66%)			0.00642	ko04712
Phenylalanine metabolism			61 (1.66%)			0.03460	ko00360
Arginine and proline metabolism			50 (1.36%)			0.04841	ko00330
Regulation of autophagy			31 (0.85%)			0.02856	ko04140
Beta-Alanine metabolism			26 (0.71%)			0.00263	ko00410
Brassinosteroid biosynthesis			15 (0.41%)			0.00975	ko00905

*Pathways with Q ≤ 0.05 were regard as significantly enriched pathways in DEGs*.

### Differential expression of signal transduction pathway transcripts

Signal transduction is a primary process for plant root development. The mitogen-activated protein kinase (MAPK) was reported to play a role in regulating the cell cycle and developmental processes (Yao et al., 2013). In this study, a total of 18 DETs showed high similarity with signaling-related genes (Table S6). Of these, two transcripts (Unigene22552 and CL14032.Contig1) encoded MAPK16 and MAPK2 were up-regulated in expanding stage; while some others including MAPK3, MPK15, and MAPK17 were down-regulated in cortex splitting stage. Calcium-regulated transduction pathway is another key signaling transduction category in cell development (Yao et al., 2013). In all, 52 DETs were homologous with calcium signaling-related genes, including 23 calcium-dependent protein kinase (*CDPKs*) transcripts, 21 calcium binding protein (*CMLs*) transcripts, five calmodulin-binding transcription activator (*CAMTAs*) and two Ca^2+^-transporting ATPase transcripts. Among them, the majority of *CAMTAs* were both up-regulated in cortex splitting stage and expanding stage; while most of *CDPKs* were down-regulated in cortex splitting stage. These results indicated that the calcium-regulated signaling pathway is a complex synergistic effect during radish taproot thickening.

In this study, a total of 614 DETs were identified to show high similarity with many plant hormone signaling pathways related genes, e.g., *AUX/IAA, TIR1, ARFs, GH3* and *SAUR* (Table S6). In detail, 396 down-regulated and 215 up-regulated transcripts were identified in the L1 vs. L2 comparison, while 334 down-regulated and 266 up-regulated transcripts were identified in the L2 vs. L3 comparison. Interestingly, six types of transcriptionally regulated genes (*AUX/IAA, GH3, SAUR, A-ARR, TCH4* and *CYCD3*) were identified in tryptophan metabolism, zeatin biosynthesis and brassinosteroid biosynthesis signaling pathways (Figure S1), which might directly relate to radish taproot thickening through influencing processes such as cell division and enlargement and plant growth. Out of these, the majority of plant hormone related genes involved in three biosynthesis and metabolism pathways showed high expression in pre-cortex splitting stage, while type-A *Arabidopsis* response regulator (*A-ARR*) family genes (11 transcripts) were up-regulated in L1 vs. L2 comparison and showed high activity at cortex splitting stage.

### Differential expression of transcription factor transcripts

At some developmental stages or cellular processes, certain families of transcription factors (TFs) may play crucial roles (Riechmann and Ratcliffe, 2000). In this study, a total of 495 DETs were identified to have high similarity with 25 TF families (Table S7; Table 3), such as APETALA2 gene (*AP2*), NAC-domain containing protein genes (*NACs*), Basic Helix-loop-helix transcription factor genes (*bHLHs*), ethylene-responsive factor gene (*ERF*), MYB domain protein genes (*MYBs*), and nuclear transcription factor Y subunit A gene (*NF-YA*). These transcripts of encoding TFs were up or down-regulated in three comparisons, implying that they might regulate the radish taproot thickening. For example, 23 *ARF* gene transcripts, 11 of them were down-regulated and 12 of them were up-regulated in L1 vs. L2 comparison. Moreover, the expression abundance of up-regulated TFs in three comparisons were also analyzed, including 10 *CAMAT* gene transcripts, *MYB* and *ARF* family which contained eight transcripts each, *NAC* and Zinc Finger (*C3HC4*) family contained seven transcripts each, six transcripts of *WRKY* family, three transcripts of *ERF* family, *bHLH, SPL* and *GRAS* family contained two transcripts each, and ARR, B3, C2H2, MADS, HSP, NF-YA and TGA family contained one transcript each. Up-regulation of these TF genes at cortex splitting and expanding stage suggests that it could positively regulate thickening taproot. In contrast, eight families (AP2, AUX/IAA, bZIP, BEE, GATA, R2R3-MYB and TCP) showed mostly down-regulation in three comparisons, implying that these TFs might play major roles in pre-cortex splitting stage.

**Table 3:** **Statistics analysis of TF gene expression during radish taproot thickening**.

**Transcription factor family**	**L1 vs. L2 (transcript) Up-or down-regulated**		**L1 vs. L3 (transcript) Up-or down-regulated**		**L2 vs. L3 (transcript) Up-or down-regulated**	
	**Up**	**Down**	**Up**	**Down**	**Up**	**Down**
AP2	1	13	14	0	4	10
ARF	12	11	15	10	17	8
ARR						
B-ARR	12	12	9	15	8	16
A-ARR	11	1	10	2	4	8
AUX/IAA	6	38	3	41	18	15
B3	4	1	3	2	1	4
bHLH	13	20	11	12	11	20
bZIP	3	7	1	8	3	7
BEE	0	3	0	3	0	3
Zinc finger						
C2H2-type	3	11	5	9	7	7
C3HC4-type	7	6	7	6	9	3
CCCH-type	3	5	4	4	4	4
B-box-type	1	4	4	1	4	1
C3H4 type	2	10	2	10	12	0
CAMTA	15	5	15	5	13	7
ERF	17	30	15	32	16	30
GATA	0	9	1	8	4	5
GRAS	5	13	8	10	9	8
MADS	2	5	5	2	6	1
MYB	18	27	16	29	18	23
HSP	3	10	6	9	9	5
NAC	16	21	9	28	16	18
NF-YA	3	1	4	0	2	2
NF-YB	0	4	1	3	4	0
R2R3-MYB	0	8	0	8	5	3
SPL	4	1	4	1	2	3
TCP	4	10	1	13	1	13
TGA	1	3	1	3	3	1
WRKY	8	28	9	27	16	19

### Differential expression of cell cycle and cell wall metabolism transcripts

The size of plants and organs is driven by cell number and cell size (Guo and Simmons, 2011). While, the key determinant of cell division rate, cell number and size is cell division cycle (Polyn et al., 2015). Interestingly, a total of 38 transcripts homologous to genes associated with cell cycle were observed as differentially regulated during radish taproot thickening in this study (Table S8), including cell division protease (ftsHs), cell division cycle 5-like protein (CDC5), cell division control protein 48-A (CDC48A), cyclin-dependent kinases (CDKs), cyclin-dependent kinase inhibitor (CDKIs), cyclin-dependent kinases regulatory subunit 2 (CKS2), cyclin (CYCs) and retinoblastoma-related protein 4 (RBR). Among these, five transcripts encoding ftsH-3, CDC48A, CYCT1-5 and RBR4 were up-regulated in all three comparisons. In addition, cell wall not only strengthens the plant body, but also has key roles in plant growth, cell differentiation, cell expansion, intercellular communication, water movement and defense (Cosgrove, 2005; Paque et al., 2014). In the present study, a total of 89 transcripts encoding the key enzymes that are involved in the cell wall synthesis and degradation, such as cellulose synthase (CESA), UDP-glucosyltransferase (UGT) and caffeoyl-CoA O-methyltransferase (CCOAMT), endoglucanase (EG), pectin lyase, glycoside hydrolase, cellulase, Xyloglucan endotransglucosylase/hydrolase (XTH) and pectinesterase (PE), were differentially regulated during radish taproot thickening (Table S8). These differentially regulated genes involved in cell wall metabolism, were also observed in sweetpotato storage root formation (Desai, 2008). Moreover, Arioli et al. (1998) showed that the cellulose synthase homolog A gene (*AtCESA*) deletion caused abnormal expansion of *Arabidopsis* roots. Taken together, these results indicate that the control of cell expansion requires cell wall metabolism. In additional, 141 transcripts encoding many other regulators of cell number and size were also found differentially regulated during radish taproot thickening in this study (Table S8), including ankyrin repeat-containing protein (AKR2), protein COBRA (COB), fasciclin-like arabinogalactan protein (FLAs), extensins (EXs), kinesins, syntaxin (STXs), aquaporin (AQP), plasma membrane aquaporin (PAQs), leucine-rich repeat family protein (LRR), proline-rich family protein (PRP) and expansin protein (EXPs). Among these, four transcripts encoding STX32, kinesin family member 2/24, PAQ2 and COB were up-regulated in all three comparisons.

### Differential expression of carbohydrate and storage metabolism transcripts

The major constituents of storage compounds in radish are carbohydrates and storage proteins, which are deposited in the developed sink tissues. In this study, 46 DETs were homologous with starch and sucrose metabolism related genes, including starch synthase genes (*SS*), ADP-glucose pyrophosphorylase genes (*ADPG-PPase*), beta-amylase, sucrose synthase genes (*SuSy*), sucrose phosphate synthase genes (*SPS*) and invertase genes (*INV*) (Table S8). Among them, the majority of *SPS* (five transcripts), *SS* (three transcripts), beta-amylase (three transcripts), and *ADPG-PPase* (one transcript) were up-regulated in expanding stage comparing with pre-cortex and cortex splitting stage. Meanwhile, 22 transcripts encoding seed storage 2S albumin-like protein and PATATIN-like protein (PLPs), were also found to be involved in storage metabolism. In addition, this study also found 36 transcripts encoding nitrite reductase (*nirK*), nitrate transporter, ATP sulfurylase (APS), sulfotransferase, sulfate transporter and sulfur E2, were involved in substances and energy metabolism. All these results suggest that many functional genes involved in radish taproot thickening regulatory networks were successfully identified in this study.

### The posttranscriptional regulatory networks of miRNAs and target mRNAs

**Figure 5:**
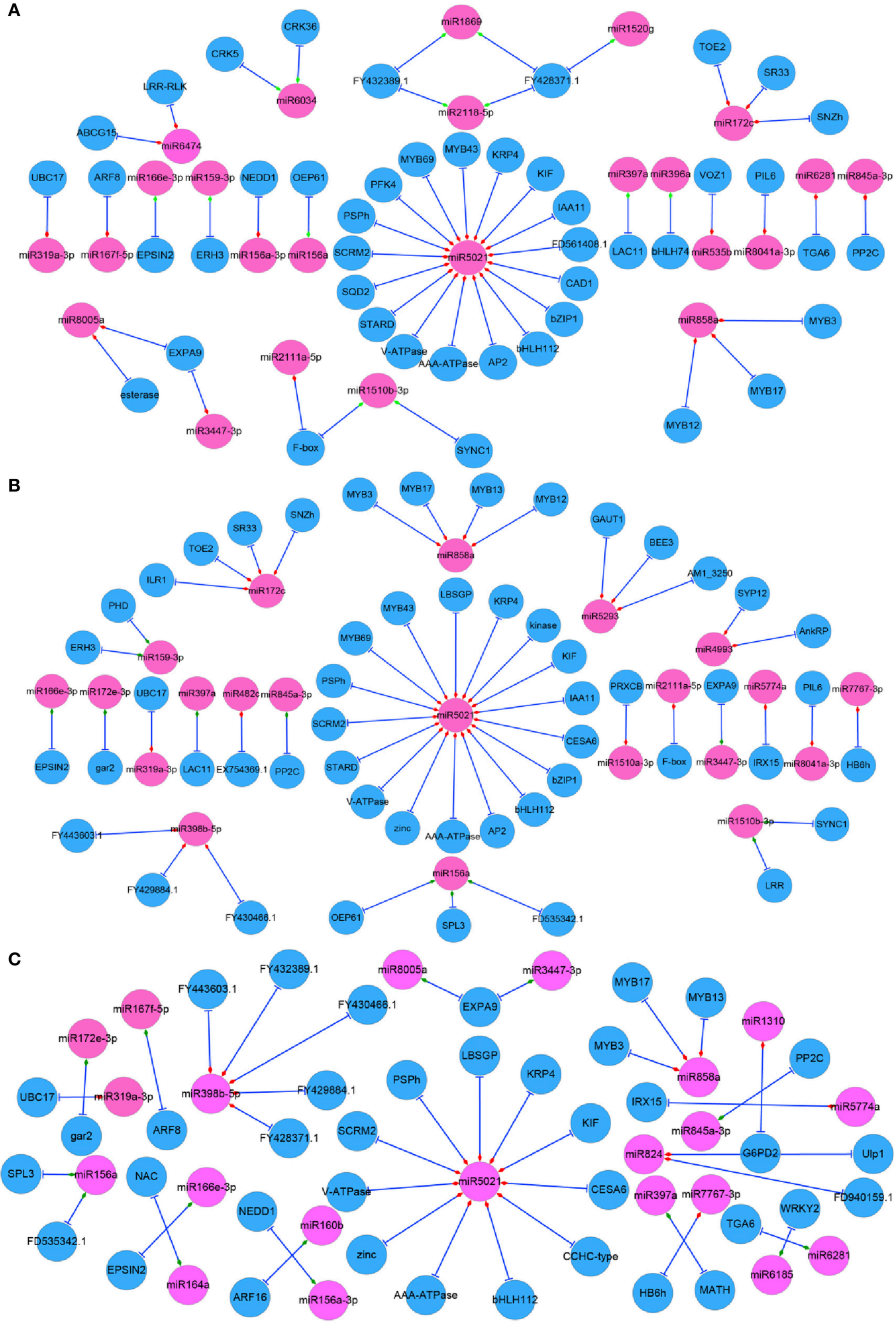
**Regulatory network from integrated analysis of miRNA–mRNA data**. Negatively correlated miRNA–mRNA interactions were visualized as a network using Cytoscape. Diamonds represent the expression type of miRNAs. Red represents up-regulation and blue represents down regulation in each comparison. **(A)** L1 vs. L2; **(B)** L1 vs. L3; **(C)** L2 vs. L3.

To fully understand the behavior of complex regulatory networks in radish taproot thickening, correlation analysis was performed based on the DEGs in the present study and taproot thickening related DEmiRs previously identified (Yu et al., 2015). Results showed that 43 miRNAs and 92 genes formed 114 miRNA-target mRNA pairs were co-expressed in three different growth stages of taproot thickening (Table S9). Among these miRNA-target mRNA pairs, a total of 24 miRNAs and 45 genes formed 55 miRNA-target mRNA pairs with negatively correlated expression in L1 vs. L2 comparison; 21 miRNAs and 52 genes formed 56 miRNA-target mRNA pairs with anti-correlated expression in L1 vs. L3; 21 miRNAs and 38 genes formed 40 miRNA-target mRNA pairs with inversely correlated expression in L2 vs. L3 (Figure 5).

Furthermore, based on GO and pathway analysis, some of target genes of differentially expressed miRNAs were annotated as transcription factors, such as squamosa promoter-binding-like protein gene (*SPLs*, targeted by miR156), MYB genes (targeted by miR858 and miR5671), *bZIP1* (targeted by miR5021), bHLH genes (targeted by miR396 and miR5021), *AP2* (targeted by miR172), ARF genes (targeted by miR160) and auxin-responsive protein IAA11 gene (*IAA11*, targeted by miR5021). On the other hand, some target genes were revealed to be involved in metabolism processes, such as 6-phosphofructokinase 4 and cellulose synthase 6.1 catalytic subunit (*PFK4* and *CESA6*, targeted by miR5021), ATP sulfurylase (*APS*, targeted by miR395) and beta-amylase 4 (*BAM4*, targeted by miR5293) (Table S9). Finally, some genes were annotated as other functional proteins, such as LRR/LRX (targeted by miR1510 and miR6474), laccase (*LACs*, targeted by miR397), Cyclin-D3-1 (*CYCD3;1*, targeted by miR6281), cyclin-dependent kinase inhibitor 4 (*KRP4*, targeted by miR5021), katanin (*ERH3*, targeted by miR159), Expansin A9 (*EXPA9*, targeted by miR8005 and miR3447) and Ankyrin repeat family protein and Syntaxin_121 (*AnkRP* and *SYP121*, targeted by miR4993) (Table S9).

**Figure 6:**
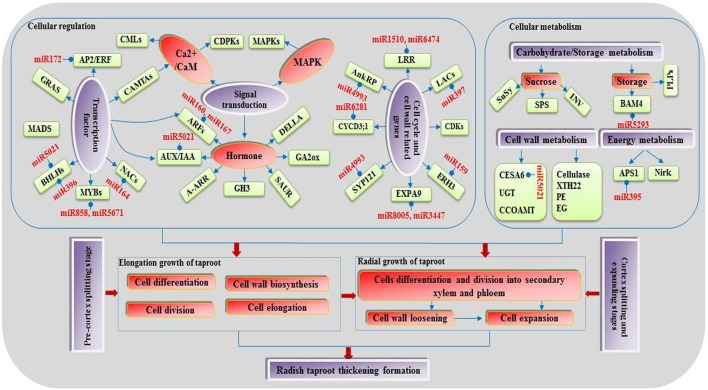
**A proposed model of genetic and molecular interactions in the regulatory network during radish taproot thickening**.

A large number of the above target genes and corresponding DEmiRs were revealed to be involved in cell events, regulation of plant hormone levels, and many signal transduction and metabolisms, including cell division, differentiation and expansion, auxin signaling, sucrose metabolism and energy metabolism, implying that they may play crucial roles during the taproot thickening process. Through the integrating analysis of the miRNA-target gene interaction and the DEGs identified in this study a complex regulatory network associated with taproot thickening and formation in radish was built in Figure 6.

### Validation of the DGE data using RT-qPCR

**Figure 7:**
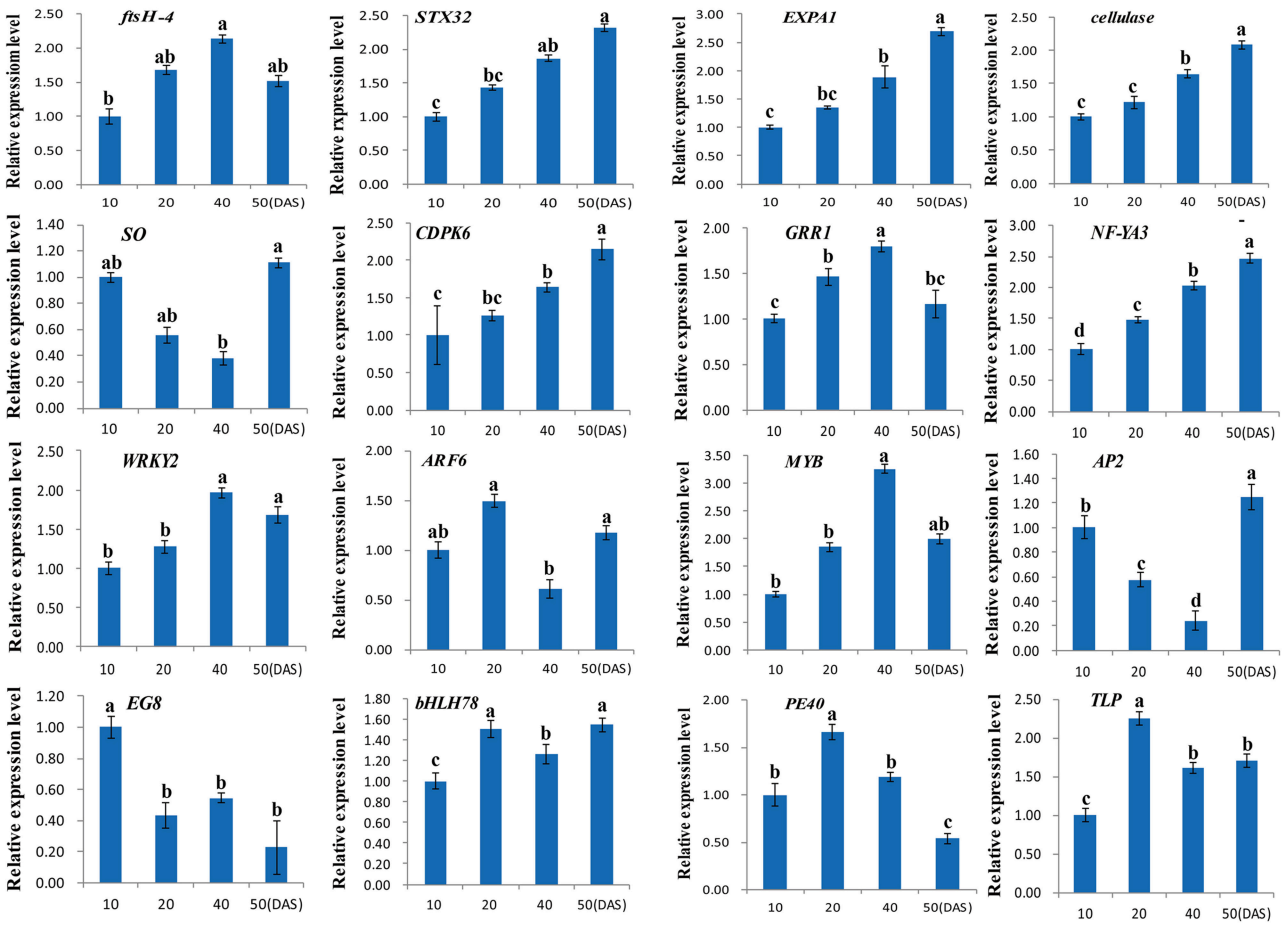
**Quantitative expression analyses of 16 DEGs in four different taproot thickening stages (10, 20, 40, and 50 DAS)**. Each column represents an average of three replicates, and each bar shows the mean ± SE of triplicate assays and an average of three replicates. The values with different letters indicate significant differences at *P* < 0.05 according to Duncan's multiple range tests.

To evaluate the reliability and validity of our DGE data, a total of 16 DEGs were randomly chosen and validated by RT-qPCR analysis (Table S1). As shown in Figure 7, all 16 DEGs were obviously differentially expressed in four different taproot development stages (10, 20, 40 and 50 DAS). Of these, the expression of 11 DEGs were up-regulated, and two DEGs were down-regulated, while the remaining DEGs showed different expression levels among the three different taproot development stages. Overall, these results indicate that 15 out of 16 DEGs showed very similar patterns as identified from DGE analysis, and only one transcript encoding auxin response factor 6 (ARF6, CL1404.Contig1_NAU-YH) was not properly consistent with the results of the sequencing.

## Discussion

Radish is one of the most marketable root vegetable crops. In plants, the formation of organs is based on successive gene expression during development, and the expression of genes also depends on specific tissues or developmental stages (Cheng et al., 2013; Park et al., 2014). Therefore, profiling of spatial-temporal gene expression is essential for elucidating the molecular mechanisms of radish taproot thickening and formation. More recently, the gene expression analysis of root development have been studied by RNA-Seq technology in some plant species, such as cucumber (Zhang et al., 2014a), lotus (Cheng et al., 2013). However, no study on comprehensive identification of the DEGs during radish taproot thickening has yet been conducted in radish to date. In this study, using RNA-Seq technology, more than seven million clean reads were generated per library and 18,144 transcripts were identified as significantly differentially expressed during radish taproot thickening. To our knowledge, this is the first study to investigate the dynamical transcriptional changes underlying radish taproot thickening. Moreover, the results in this study implied that these DETs are likely to be involved in the regulation of radish taproot thickening and formation.

### Potential DEGs playing critical roles for radish taproot thickening and formation

The thickening of radish fleshy taproot is a complex biological process involving morphogenesis and metabolite production accumulation. This process results from the thickening of the hypocotyl and primary root (Tsuro et al., 2008), which is mainly due to the activity of a vascular cambium and gives rise to secondary xylem and phloem. In addition, the growth of secondary xylem and phloem, especially in xylem parenchyma cells development, depends on the cell differentiation, division, and expansion in these regions and results in the rapid increase of root diameter, which is driven by different regulatory factors including plant hormones, transcription factors, and many metabolism pathways etc. (Choi et al., 2011; Ursache et al., 2013).

#### Hormonal signaling regulation

The plant hormones (i.e., auxin, cytokinin, gibberellin, ethylene, jasmonate and brassinosteroid), have been found to be important signals in plant root development (Jung and McCouch, 2013; Ljung, 2013) including root apical meristem (Benková and Hejátko, 2009), root architecture (Overvoorde et al., 2010). Additionally, many studies have indicated that the hormone-related genes can be involved in the secondary growth of cambium by regulating cell division, differentiation and expansion (Dolan and Davies, 2004; Korasick et al., 2013; Ursache et al., 2013; Kong et al., 2014). In the present study, based on GO and pathway annotation, the plant hormone-mediated signaling pathway was the most enriched one. A total of 614 DETs were identified to show high similarity with many plant hormones signaling pathways related genes, which are involved in eight biosynthesis and metabolism pathways (Table S6). Among those hormone related genes, auxin has been shown to modulate cell proliferation and cell expansion in part by changing gene expression (Qiu et al., 2013; Ursache et al., 2013). Among three primary auxin-induced gene families (GH3, AUXI/IAA and SAUR), GH3 protein families have been shown to be involved in hypocotyl and root development (Takase et al., 2004). Zhang et al. (2014b) revealed that the auxin response factors ARFs were down-regulated in the mutant Aux/IAA protein RUM1, which controls the differentiation of vascular cells, and the Aux/IAA protein RUM1 gene expression in maize primary roots. In this study, most transcripts encoding Aux/IAA protein genes were down-regulated, while those encoding ARFs genes were up-regulated at the cortex splitting and expanding stage, implying that they may be involved in the cambium secondary differentiation during radish taproot thickening. SAUR transcripts or SAUR proteins have been proposed to promote cell proliferation and cell expansion in root development by unknown pathway (Qiu et al., 2013; Kong et al., 2014). In this study, the expression of two SAUR transcripts (EW714406.1 and Unigene14134_NAU-YH) were up-regulated during taproot thickening, implying that they may relate to the cell expansion in the secondary growth of cambium.

In addition, many researches have testified that other hormones such as cytokinin, gibberellic acid (GA), jasmonic acid (JA), abscisic acid (ABA), brassinosteroid (BR) and ethylene are also involved in the regulation of storage organ formation and the secondary tissue development (Fernández Calvo et al., 2011; Rayirath et al., 2011; Abelenda and Prat, 2013; Ursache et al., 2013). The transcripts of type-A ARR genes, which have been reported to be rapidly induced upon cytokinin treatment of plants (Kiba et al., 2003), were mostly up-regulated in the present study. Moreover, Fu and Harberd (2003) reported that GA, auxin and ethylene effect cell growth in the root by opposing the action of DELLA proteins. Overall, these results suggest that these hormone signals related genes play vital roles in taproot thickening in radish.

#### Up-regulation of transcription factors during taproot thickening

All major processes of life depend on differential gene expression, which is largely controlled by the activity of transcription factors (Montiel et al., 2004; Zhang et al., 2014a). In the current study, we detected many transcription factors that were differentially expressed in the three samples (Table S7). Of these, 65 unique transcripts encoding 16 transcription factor families were significantly up-regulated during radish taproot thickening (Table S7). For these transcription factors, several transcription factors including MADS (Unigene16007), CAMTA (Unigene22158, CL9027.Contig2 and Unigene1675) and bHLH (Unigene11149, EY929981.1) have been identified to perform critical roles in the formation of underground storage organ (You et al., 2003; Fernández Calvo et al., 2011; Yao et al., 2013; Chiasson et al., 2014). In sweetpotato, two isolated MADS-box protein genes (*IbMADS3* and *IbMADS4*) were mainly expressed in root tissues (Kim et al., 2002), and found in the vascular cambium region where the most active cell proliferation occurs during storage root development (You et al., 2003). Moreover, Ku et al. (2008) showed that MADS-box 1 (IbMADS1) is an important integrator at the onset of storage root formation. Calmodulin (CaM) is a sensor of Calcium ions (Ca^2+^), Ca^2+^ is a crucial regulatory ion in cell expansion (Dolan and Davies, 2004), and Ca^2+^/CaM and CaM-bingding proteins have been found to be involved in the formation of storage organs (Balamani et al., 1986; Cheng et al., 2013; Yao et al., 2013).

Furthermore, some transcripts encoding MYB (CL8915.Contig3, Unigene26378 and CL6324.Contig1), NAC (CL8699.Contig1), and GRAS (EX888303.1) families were up-regulated during taproot thickening (Table S7), which play key regulatory functions in cell differentiation, division and expansion, as well as the cambium and secondary tissue development. It was reported that MYB and NAC TFs family might control secondary cell wall metabolism (Mitsuda et al., 2005; Zhong et al., 2007, 2011). Meanwhile, some NAC TFs family have also been shown to be involved in the development of secondary xylem (Ohashi Ito et al., 2010; Han et al., 2012), and regulating the transition from growth by cell division to growth by cell expansion (Sablowski and Meyerowitz, 1998). In *Arabidopsis*, the GRAS TF family member SCARECROW (SCR) was expressed in cortex/endodermal initial cells in root system playing key role in regulating the radial organization of the root (Di Laurenzio et al., 1996). All these studies suggest that these TF genes may be potentially involved in the radish taproot thickening.

#### Carbohydrate and storage metabolism

Starch is considered as one of the major storage carbohydrates. In this study, starch biosynthesis related starch synthase (Unigene17497, CL2179.Contig3 and Unigene29292) and ADP-glucose pyrophosphorylase (Unigene650) were up-regulated at the three developmental stages. Similarly, three transcripts (CL1841.Contig1, CL2019.Contig1 and Unigene1147) encoding beta-amylase, starch degradation related gene, were also up-regulated in three libraries. This finding is similar to that of Takahashi et al. (2012) and Hara et al. (2009) in which beta-amylases activity was elevated in the growing taproot of the radish, where starch content increased, implying that beta-amylases may be storage proteins in plant taproot (Gana et al., 1998).

Sucrose is the major product of photosynthesis, which plays an important role during the formation of storage organs (Usuda et al., 1999). In radish, the storage root is a major sink, which begins to thicken early in development. In this study, some transcripts encoding genes including sucrose synthase (*SuSy*), sucrose phosphate synthase (*SPS*) and invertase (*INV*) were found to be associated with the sucrose metabolism (Table S8). The similar results were obtained in the previous study, and the SuSy, INV and SPS were encoded by the higher numbers of transcripts in annotated ‘NAU-YH’ root transcriptome dataset (Yu et al., 2016). Evidence shows that the import and accumulation of sucrose in storage roots might involve its inversion into hexose sugars by invertase and sucrose synthetase (Ruan, 2014). Rouhier and Usuda (2001) and Usuda et al. (1999) showed that SuSy is a key enzyme in the early development of the storage root of radish. The transcripts encoding *SuSy* (FD545600.1, EY948143.1 and EY943900.1) and *INV* (Unigene22581, Unigene22582, EY934184.1 and FD531565.1) were mostly down-regulated during taproot thickening in this study, implying that they play an important role at the initial thickening stages of the taproot. Furthermore, Jackson (1999) showed that high content of sucrose is required as a necessary condition during the formation of storage organs. In the present study, transcripts encoding *SPS* (CL10532.Contig1, CL6177.Contig1, CL13324.Contig2 and Unigene14135), which is the major source of sucrose synthesis activity (Ren and Zhang, 2013), were mostly up-regulated during taproot thickening, implying it may play a major role in the taproot expanding stage.

Patatin and 2S albumins are generally considered as storage proteins. Evidence shows that patatin might be associated with the formation of underground storage organs based on its expression profile (Stupar et al., 2006; Cheng et al., 2013). In the present study, transcripts encoding patatin and 2S albumins proteins were mostly highly expressed in L2 library, which obviously showed that storage proteins might have some relationship with radish taproot thickening.

### The regulatory networks associated with radish taproot thickening

Root development and response to the environment are thought to be controlled by gene regulatory networks (Petricka et al., 2012). miRNA-mediated gene regulation at transcriptional and posttranscriptional levels has been extensively studied in root development in other plant species (Liu et al., 2013; Lakhotia et al., 2014), and the roles of some miRNAs were also identified in root development (Khan et al., 2011; Bustos Sanmamed et al., 2013), which greatly advanced our understanding of the molecular regulatory networks underlying the root thickening process. In this study, a putative model of regulatory network associated with radish taproot thickening and formation was proposed according to our transcriptomics analysis and previous research achievements (Figure 6). During the vegetative growth, some environmental factors such as temperature, light and soil fertility stimulate the first and second vascular cambia initiation. The cells take up energy or nutrients through some signal transduction pathways (hormone, calcium and MAPK signaling) and metabolism possesses (cell wall, carbohydrate, storage and energy metabolism). Some regulation factors promote cell differentiation, division and expansion at the secondary xylem and phloem. In this study, some miRNA-mRNA pair interactions were observed in radish taproot thickening process. In detail, the *ARFs* (targeted by miR160 and miR167), *IAA11* (targeted by miR5021) and bHLH74 (targeted by miR396) were found to be involved in root growth and regulated vascular cell differentiation (Bustos Sanmamed et al., 2013; Bao et al., 2014; Zhang et al., 2014b). The LRR protein kinase-like protein gene (*LRR*, targeted by miR1510 and miR6474), *LACs* (targeted by miR397) and *EXPA9* (targeted by miR8005 and miR3447) were involved in cell wall formation and loosening (Dolan and Davies, 2004; Cai et al., 2006), while the *CESA6* (targeted by miR5021) and *BAM4* (targeted by miR5293) genes were involved in cell wall synthesis and degradation (Cosgrove, 2005; Van Sandt et al., 2007). In addition, *ERH3* (targeted by miR159), NAC-domain protein gene (targeted by miR164), *AnkRP* (targeted by miR4993) and *KRP4* (targeted by miR5021) were involved in cell differentiation and division (Zhao et al., 2005; Desai, 2008; Tominaga Wada and Wada, 2014). The findings indicated that some miRNA-mediated gene interactions were existed among these DETs in the cell wall formation and differentiation process. Moreover, some metabolism processes also play key roles in radish taproot thickening regulation. The *SuSy* gene was correlated with root thickening rate in carbohydrate metabolism process (Mitsui et al., 2015), while the *APS1* (targeted by miR395) was involved in root elongation regulation of sulfur metabolism process (Zhao et al., 2014). Taken together, the results implied that all these DETs could play important roles in the regulatory network of radish taproot thickening and formation. After the further functional validation, these critical genes would greatly contribute to manipulate the radish taproot shape, yield and quality.

## Conclusions

In summary, a total of 18,144 transcripts that were differentially expressed among different taproot thickening stages were firstly identified using DGE technology. GO and KEGG pathway enrichment analysis revealed that these DETs were mainly involved in processes of cell events, metabolism, biosynthesis and signaling transduction pathways. Furthermore, the integrated analysis of mRNA-miRNA revealed that the miRNA-mediated gene regulation had a dramatic impact on the taproot thickening of radish. A hypothetical model of genetic regulatory network associated with taproot thickening in radish was put forward. The taproot formation of radish is mainly attributed to cell differentiation, division and expansion, which are regulated and promoted by certain specific signal transduction pathways and metabolism possesses. These findings could not only accelerate the process of genetic improvement of taproot in radish, but also provide novel insights into the molecular regulatory mechanism underlying taproot thickening and formation in root vegetable crops.

## Author contributions

All authors listed, have made substantial, direct and intellectual contribution to the work, and approved it for publication.

### Conflict of interest statement

The authors declare that the research was conducted in the absence of any commercial or financial relationships that could be construed as a potential conflict of interest.

## Supplementary material

Figure S1 **The DEGs involved in pathway of plant hormone signal transduction. (A)** DEGs between L1 and L2 library; **(B)** DEGs between L1 and L3 library; **(C)** DEGs between L2 and L3.

Table S1 **The primers of 16 DEGs validated by RT-qPCR analysis**.

Table S2 **A set of 18,144 differentially expressed transcripts in three comparisons**.

Table S3 **The analysis of DEGs with an RPKM value ≥ 100 in at least one of the three comparisons**.

Table S4 **GO enrichment analysis for 18,144 differentially expressed transcripts**. GO terms with corrected *p* ≤ 0.05 **(A)** and *p* ≤ 0.001 **(B)** were regarded as significantly enriched ones.

Table S5 **Overview of all 127 KEGG pathways for DEGs in three libraries based on pairwise comparison**.

Table S6 **The differentially expressed transcripts involved in root thickening-related signaling pathways**.

Table S7 **The differentially expressed transcripts encoding transcription factors during radish taproot thickening**.

Table S8 **The differentially expressed transcripts involved in cellular process and metabolism during radish taproot thickening**.

Table S9 **The correlation analysis between DEGs and DEmiRs during radish taproot thickening**.
